# Acceptability of Stool-Based DNA Colorectal Cancer Screening among Black/African-American Patients Served by Federally Qualified Health Centers

**DOI:** 10.1007/s13187-025-02631-0

**Published:** 2025-05-01

**Authors:** Evan Keiser, A. Michelle Corbett, Onyema Chido-Amajuoyi, Allison Antoine, Carrie Stehman, Isabella Dorn, David Goines, Noelle K. LoConte

**Affiliations:** 1 School of Medicine and Public Health, Department of Medicine, University of Wisconsin, Madison, WI USA; 2 Center for Urban Population Health, School of Medicine and Public Health, University of Wisconsin, Milwaukee, WI USA; 3 University of Washington, Fred Hutch Cancer Center, Seattle, WA USA; 4 Outreach Community Health Center, Milwaukee, WI USA; 5 Progressive Community Health Centers, Madison, WI USA; 6 University of Wisconsin Carbone Cancer Center, Madison, WI USA

**Keywords:** Colorectal cancer, Screening, Disparity

## Abstract

Colorectal cancer (CRC) has an increased burden among Black/African-American populations. Following the COVID-19 pandemic, home-based CRC screening options are being used more frequently. We conducted focus groups to understand the acceptability of stool-based DNA testing for CRC screening in this population. Ten focus groups about the acceptability of various CRC screening modalities were held with Black/African-American participants at two federally qualified health centers (FQHCs) in Milwaukee, Wisconsin. Participants were separated into focus groups based on age and gender. Thematic analysis was carried out using NVivo. Across the groups, there were a total of 79 participants, of which 40.5% were aged 40–50 years (“younger participants”), 59.5% aged > 50 years (“older participants”), 53.2% male, and 46.8% female. Overall, knowledge was low regarding perceived risk of CRC. There was limited awareness of CRC screening options among younger patients and widespread lack of knowledge about stool-based DNA testing. Most respondents preferred colonoscopy as their first-choice screening test but were open to other screening tests. Stool-based DNA tests were more preferred among younger participants but was felt to be acceptable across all groups. Given the low awareness/knowledge of screening modalities identified in our study, educational interventions and shared decision making by primary care providers are needed.

## Introduction

In the United States (US), colorectal cancer (CRC) has a high morbidity and mortality, projected to be third out of all malignancies in 2023 [1]. Based on these risks, the US Preventative Services Task Force recommends all average-risk adults aged 45–75 years be screened for CRC, with the minimum age decreased to 45 from 50 in 2021. There are seven recommended screenings by the USPSTF: fecal immunochemical testing (FIT) every year, stool-based DNA testing every 1 to 3 years, colonoscopy screening every 10 years, computed tomography colonography every 5 years, flexible sigmoidoscopy every 5 years, flexible sigmoidoscopy every 10 years + annual FIT, or high-sensitivity guaiac fecal occult blood (HSgFOBT) testing every year [2]. More recently in 2024, the FDA also approved a blood-based cell-free DNA screening test for CRC [3]. In 2021, a National Health Interview Survey evaluated the usage of different CRC screening modalities, which showed a higher usage of colonoscopy (57%) compared to stool-based tests (11%) in Black populations [4]. However, these surveys have not specifically looked at comparing the different stool-based tests such as FIT or stool-based DNA [4].

Among the recommended CRC screening modalities, each detects cancer in different ways. FIT is a non-invasive method that uses an assay of antibodies to detect human hemoglobin in stool [5]. Stool-based DNA screening is also non-invasive and includes a FIT with added detection of abnormal DNA molecular biomarkers present in cancer (mutant KRAS, methylated BMP3, methylated MDRG4) [6]. Colonoscopy visually examines and removes concerning lesions in the rectum and colon [7]. Colonoscopy has been utilized for significantly longer than FIT or stool-based DNA testing, with approval for colonoscopy in 1998 versus the latter two in 2014 [8, 9].

The American Cancer Society has evaluated CRC screening rates by race from ages 45 to 75 as recently as 2021. Rates were 61%, 61%, 52%, 52%, and 50% for Black, White, American Indian/Alaska Native, Hispanic, and Asian/Pacific Islander populations, respectively [10]. However, Black Americans have increased rates of CRC compared to other populations. In 2023, there was 33% higher mortality and 15% higher incidence of CRC in Black compared to White Americans [11]. Some known causes of this inequity for Black Americans include structural racism, individual lifestyle choices, access to quality treatment, and medical mistrust, among many more [12–17]. These inequities often lead to suboptimal CRC screening rates and presentation with advanced disease [15, 16].

Given the higher mortality rate of CRC among Black/African Americans, as well as a need to boost screening rates in this population, we studied the acceptability, interests, and drawbacks of stool-based DNA testing among Black/African-American volunteers. To help understand the causes of gaps in screening, we sought out opinions and hesitations about different CRC screening methods. A particular emphasis was placed on assessing awareness and subjective acceptability of stool-based DNA screening due to the novelty of the method, an increase in home-based testing since the COVID-19 pandemic, and the lack of comparison between stool-based tests acceptability in the literature.

## Methods

### Study Design/Population

Ten focus groups were held in two federally qualified health centers (FQHCs) in Milwaukee, Wisconsin from June to October 2023: Outreach Community Health Center and Progressive Community Health Centers. Participants were recruited via direct text messages from population health specialists in both FQHCs based on purposive sampling by age only. Eligibility was verified by FQHC staff who partnered with the research team to screen interested participants. No demographic information was collected besides age and gender. Occasionally participants provided their age during focus group discussion.

Participants aged 40–50 years were recruited to assess preferences in a population that had less experience with screening comparatively to aged greater than 50 years. Participants were assigned to four categories that created 10 focus groups: younger men (under 50), younger women, older men (50 and above), and older women. Of the 10 focus groups, there were three groups each of “younger men” and “younger women” and two groups each of “older men” and “older women.” Participants were unique and did not join multiple focus groups. The same facilitator, a co-author, led all focus groups for 90 min. A non-clinical staff representative (i.e., community health worker or population health specialist) assisted the facilitator during sessions. Individuals were compensated $40 at the completion of their focus group session. The study was deemed exempt from review by the University of Wisconsin Health Sciences Institutional Review Board.

### Focus Groups

At the beginning of each focus group session, participants were provided educational materials about CRC screening modalities. Furthermore, they were shown an educational video about stool-based DNA tests and asked open-ended questions (Table 1) exploring the awareness of, experience with, and opinions about each CRC screening test. The same questions were asked in each focus group. The final question of each focus group asked each participant to name their preferred screening method and to explain their choice.

**Table 1:** Questions posed to participants in focus group sessions

Focus group questions
• There are several ways to screen/test for CRC. What ways have you heard of or had experience with?
• What have you heard about stool-based DNA testing for CRC?
• Do you know anyone who has personally used it [stool-based DNA testing]?
• What were their experiences [with stool-based DNA testing]?
• What do you like about the Stool-DNA test? [Probe: Compared to colonoscopy or FIT, does anything seem better about this type of screening test [Stool-DNA/Cologuard]?
• What about the stool-DNA test don’t you like? [Probe: Compared to colonoscopy or FIT, does anything seem worse about this type of screening test [Stool-DNA/Cologuard]?
• Part of screening for CRC with a stool-DNA test involves examining your DNA. What concerns do you have about that, if any?
• Test specimens are kept at Exact Sciences for use in other research projects. What concerns do you have about that, if any?
• If you were trying to decide which CRC screening test to use (colonoscopy, FIT, Stool-DNA/Cologuard) what would you think about to help you decide which test is best for you?
• The COVID-19 pandemic impacted health care in many ways. How has the COVID-19 pandemic impacted CRC screening for you and people you know?
• If you had to choose, which screening test would you choose, and why?

### Data Analysis

The focus groups were audio recorded for transcription purposes. Thematic analysis of transcriptions was carried out using NVivo. Two coauthors independently used an iterative process of deductive and inductive coding. Initial codes were established based on the question guide, applied deductively. Additional codes were developed based on themes that emerged from the data. Agreement was reached between coauthors and presented to the entire research team to achieve broader consensus.

The characteristics of responses were characterized using descriptive statistics, with respondents stratified into age and gender. Based on responses to standardized questions and discussions, themes were created about preferred screening modality and positive/negative decision-making factors about each screening. Basic descriptive statistics were used to analyze the data around modality choice and reasoning behind it. This was subsequently displayed in the radar charts. All high-risk individuals were excluded from modality preference analysis, leading to 70 average-risk individuals being included. Out of the 70 average risk participants, 68 of them gave a response. Percentages were created based on choice, divided by total number of participants in that subgroup based on age and gender. For Fig. 2 with all 79 participants’ opinions included, there were no percentages calculated. The radar chart was a “heat map” with the number of times that a factor was cited among all participants. Each participant could name a decision-making factor once.

## Results

A total of 3314 participants were solicited for participation via text, with a response rate of 17.3%. Out of the 573 interested in participation, 79 participants were recruited to focus groups based on a purposive sample. These participants were categorized by age (younger [40–50] and older [over 50]) and gender (men vs. women). By age, there were 32 younger (40.5%) and 47 older (53.2%) participants. By gender, there were 42 men (53.2%) and 37 women (46.8%) participants.

### Screening Preference

Of the 70 average-risk participants, 68 (97%) named a preferred method. The other nine participants (11%) were high-risk based on prior colonoscopy results or familial risk, so were excluded as their risk necessitated colonoscopy screening.

**Fig. 1:**
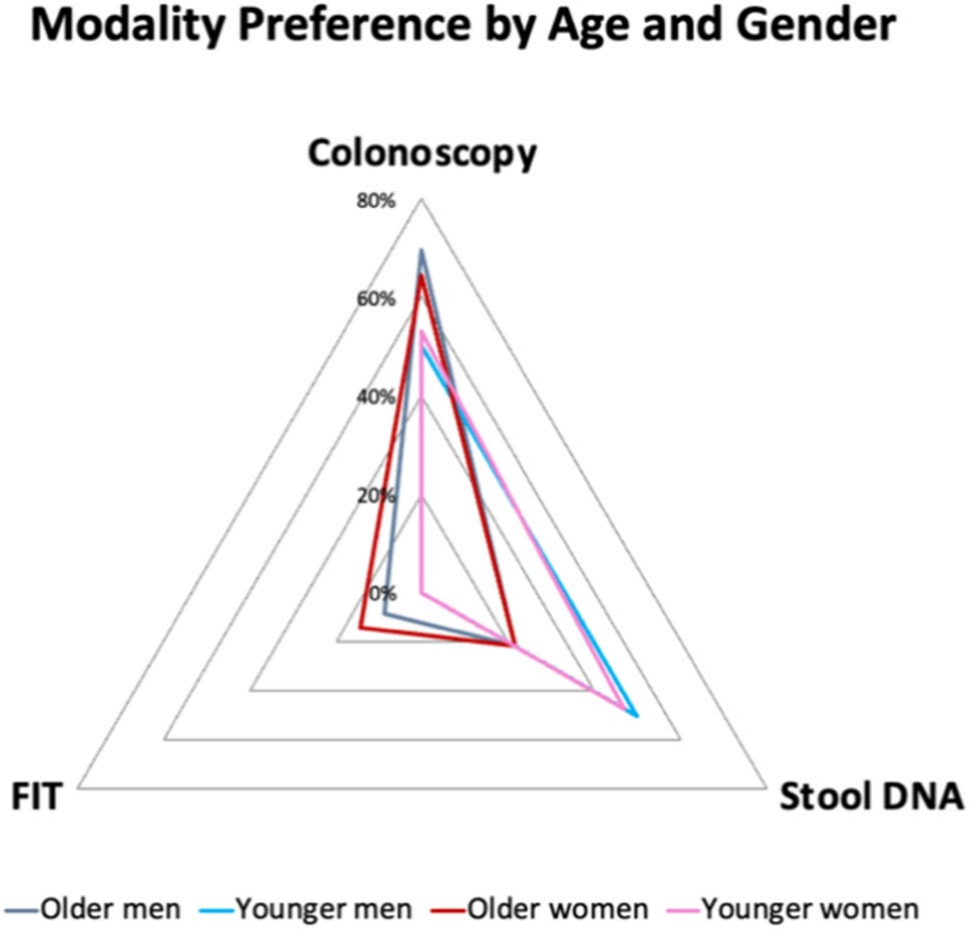
Radar chart describing the percentage of 68 total participants stating the preferred modality of CRC screening, categorized by age and gender. It excludes 11 participants out of 79 in focus groups who are at high risk and necessitating colonoscopy and those that did not pick a preference

As described in Fig. 1, colonoscopy was the most preferred screening test. This finding held true across subgroups, with younger men favoring colonoscopy at a rate of 50%, while the other 50% chose stool-based DNA. Like younger men, younger women slightly preferred colonoscopy (53%) compared to stool-based DNA testing (47%). Older men and women were the groups with the highest rate choosing colonoscopy at 70% and 64%, respectively.

Figure 1 highlights that the younger study population had a higher preference for stool-based DNA than FIT testing compared to older groups. Stool-based DNA was preferred by 22% of older men and 21% of older women. Older groups were the only participants to choose FIT as a preferred test, with older men at 9% and older women at 14%. No participant in the young study population chose FIT testing as their favored screening method.

### Decision-Making Factors

Reasons supporting the preferred screening method ranged from convenience to accuracy and differed by gender and age.

**Fig. 2:**
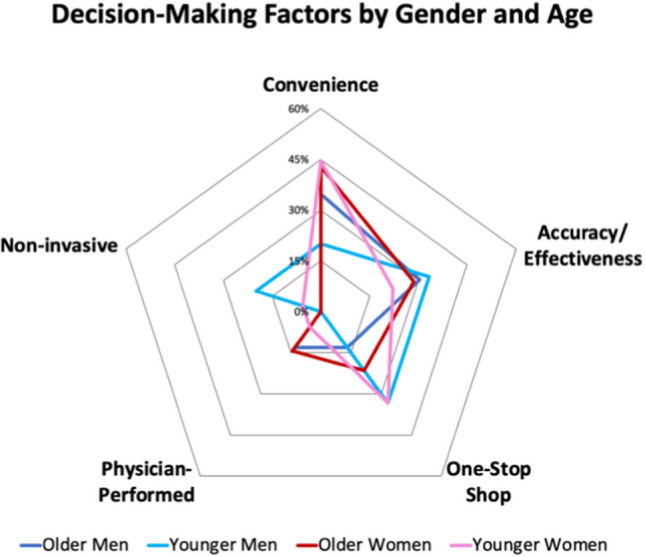
Radar chart describing the number of frequently used phrases among focus groups, categorized by gender and age

Figure 2 demonstrates the five frequently used phrases among all 10 focus groups. This figure includes opinions from all 79 participants. Participants cited more than one reason. The most cited factor in older men, older women, and young women was convenience at 35%, 43%, and 44%. In young men, accuracy/effectiveness and “one-stop shop” (or the ability to complete the screening in one occurrence) were most cited at 33% each.

Convenience was emphasized when speaking of home testing, such as FIT and stool-based DNA testing. As above, it was the most cited of all groups besides younger men (20%). Participants enjoyed the idea of screening in their own home where they could quickly complete it. An example of this was when one participant highlighted “To me, this [FIT] so simple to me. They said I could do it at home… I rather try and do the FIT test. It was easy and convenient.” This idea is opposed to colonoscopy where one would have to take time off work, doing “prep” for colonoscopy.

Accuracy/effectiveness was cited for colonoscopy and stool-based DNA. Most participants did not know prior to the session that there were differences in what the three methods tested. When the facilitator provided this information, many found this an important factor in their preferred choice, such as when one participant said, “I think it’ll [stool-based DNA] make you feel better, give you more detail, accuracy in knowing, if you do or if you don’t. This will lead to, so if you do, to colonoscopy”. Overall, accuracy/effectiveness was the second-highest cited factor, with no significant difference between men (30% older + 33% younger) and women (29% older + 22% younger).

One-stop shop was a term used by participants, along with “cutting out the middleman,” which referred to how a colonoscopy can be used to screen, remove, and diagnose in a single step. A participant highlighted this sentiment when one said, “For me, it’s better, and it’s faster, and if they can see anything, it’s right in their face, and I don’t have to send in anything for them to have to test because they’re going to do it all, right there, with one. For me, it’s a one-stop-shop.” This feature was important among those that realized at-home screening modalities could result in the need for a confirmatory colonoscopy. One-stop shop was highlighted more in younger men and women (33% + 33%) compared to older cohorts (13% + 21%), respectively.

Physician-performed and non-invasive were both in reference to colonoscopy, but for opposite reasons. Physician-performed was positive, desiring an expert to perform the screening. One participant stated, “With something that’s as serious as colon cancer, it just seems like you may actually want to go through the whole process [a colonoscopy] with a doctor.” Non-invasive was negative, preferring no procedure inside of their body, such as when one participant showed concern saying, “I’d rather do that [stool-based DNA] than have them stick anything up back there.” Twenty percent of younger men cited non-invasive being important, but 0% remarked on physician performed. Six percent of younger women and 0% of the older cohort found non-invasive important. Fourteen percent of the older cohort remarked on physician-performed being important.

## Discussion

In this study of Black/African-American patients served at FQHCs who were at average risk for CRC, stool-based DNA is generally acceptable. Some had exposure to this form of testing from commercials and direct-to-consumer advertisements, but were not aware of the accuracy, less frequent screening compared to FIT, and convenience. Many highlighted the high accuracy, convenience, privacy, and simple instructions of stool-based DNA as positive reasons for this screening modality. Questions regarding the use of DNA were levied to focus groups, but examination of one’s DNA was not a pervasive concern. A concern for this method was if the test was positive, a colonoscopy would be needed. Based on the positive subjective perceptions and benefits outweighing the drawbacks in this method of screening, it was deemed that stool-based DNA is acceptable to this population.

The overall preferred screening method was colonoscopy (60.3%), followed by stool-based DNA (33.8%) and FIT (5.9%). It is important to highlight that these preferences were ascertained later in the focus group after education had been provided. These findings are novel compared to most other studies in the literature, as most studies have evaluated preferences prior to stool-based DNA testing being approved by the FDA in 2014 [18–22]. For example, Schroy et al. in 2006 showed colonoscopy and stool-based DNA tests were preferred over FOBT, but their cohort was not stratified for a Black population [18]. Hawley et al. in 2008 did not include stool-based DNA as an option, but found in their sub-cohort, who are Black, flexible sigmoidoscopy and colonoscopy were favored compared to FIT and FOBT [19]. Shokar et al. investigated subjective feelings and preferences (not including stool-based DNA) with a Black population and found FOBT was preferred over colonoscopy [20]. However, after receiving education, preferences switched to colonoscopy over FOBT [20]. Palmer et al. performed a similarly structured study to ours, finding colonoscopy was the preferred screening method over FOBT, flexible sigmoidoscopy, and barium enema [21]. Lastly, Chablani et al. analyzed a cohort of Black and Latino participants, using focus groups that assessed preference between four screening methods, finding colonoscopy was preferred followed by stool-based DNA then others [22]. This study did not analyze the Black population separately for preference [22].

Our study highlights not only colonoscopy was preferred over stool-based DNA and FIT, but also demonstrates preferences based on age and gender for the first time in a Black population. In Fig. 1, older demographics preferred colonoscopy compared to younger participants (68% vs. 52%, respectively). An even larger difference between younger and older populations was observed in stool-based DNA (48% vs. 22%, respectively). When looking at the large difference, many felt positively about colonoscopy and stool-based DNA testing’s high accuracy. The sensitivity and specificity of these methods for detecting CRC have been well demonstrated [15]. The sensitivity and specificity of these tests are respectively: 0.89–0.95 and 0.89–0.94 for colonoscopy, 0.93 and 0.85 for stool-based DNA, plus 0.74 and 0.94 for FIT [7]. Many participants desired the modality that would rule out CRC, which deterred from FIT.

However, the younger population did feel invasiveness of a test was important compared to the older demographic (12% vs. 0%, respectively). Younger cohorts (3%) did not find “physician-performed” as important compared to older groups (13%). Preferring noninvasive testing without a physician performing the exam could be factors why the younger cohort favored stool-based DNA over colonoscopy. Interestingly, the factor of one-stop shop or “cutting out the middleman” was highlighted most frequently by the younger cohort (33%) compared to the older population (16%). This factor referred to colonoscopy, where one could have testing, removal, and diagnostics in one procedure without needing additional screening if positive on FIT/stool-based DNA.

As noted above, the most cited factor was convenience. Convenience was never mentioned in reference to colonoscopy, only FIT and stool-based DNA. As the younger populations are more likely employed, the convenience of testing at home is appealing. Compared to colonoscopy, where one misses work, arranges for transportation, and drinks preparation material the day before, stool-based DNA testing or FIT may be more feasible. This was the highest impact factor for all groups besides younger men (20%). Younger women, older men, and older women all had higher rates at 44%, 35%, and 43%, respectively.

The FIT was the least preferred test, with only 11% of the older age group choosing it. Factors impacting choice for the FIT test positively included convenience, only mentioned by the older demographic. Many other participants felt that this method was “messy” because one scoops their own stool comparatively to stool-based DNA, which is collected via a hat. The decreased accuracy also deterred participants.

Between genders, there were no consistent differences that were observed across age ranges. The only isolated difference seen was seen between younger men (20%) and younger women (44%) stating convenience as an important factor.

Throughout focus groups discussions, one recurrent subtheme was low awareness. There was minimal knowledge of one’s risk for CRC, such as the concept of average versus high risk. An example included one young woman who stated “I knew my mom went to get a colonoscopy… I really didn’t know, didn’t understand, and I got a lot of information. I really appreciate this [focus group].” Another participant in the older women group remarked about preventative screenings, saying she had no “understanding of that’s [screening] was what we deserved.”

An important additional discussion point with this population is cost though it was not a pervasive concern among participants. All three modalities are covered by insurance. Furthermore, the Affordable Care Act requires Marketplace insurance plans and most private insurance to cover CRC screening for average-risk patients of age without cost sharing/co-payment. This was discussed during groups with the facilitator when educating about the various options ($30 for FIT, $600 for stool-based DNA, and minimum $1600 for colonoscopy out of pocket). For an uninsured patient, the FIT test would be the most affordable option. Both FQHC’s in this study had an uninsured population of < 17%.

Many participants asked detailed questions about testing methods, as many had never been screened. For example, many did not know that positive FIT/stool-based DNA tests would necessitate diagnostic colonoscopies. None of the participants had experience with stool-based DNA screening. Several were appreciative of the focus groups because they “used to think all of them [CRC screenings] gave the same information.”

Given the need for education as well as the number of CRC screening tests available, we believe it is critical for shared decision making to be a priority to increase CRC screening. The American Cancer Society and Center for Disease Control and Prevention created a “80% by 2018” goal for 80% of recommended adults to be screened, but as of 2023, 67% were screened [5, 23]. On our observational findings, participants had lower awareness and chose their preference based on education, which has been demonstrated in prior studies [20]. During focus groups, participants placed emphasis on experiences by members of their community, good or bad. If we can increase education with a trusted member of the community, such as a community health worker, we may be able to increase screening for this vulnerable population through team-based care. Use of community-based navigation to increase screening has been shown before, and our study supports this claim [24, 25]. Overall, this study highlighted that while colonoscopy was preferred in this population of Black American patients, stool-based DNA testing was acceptable, and team-based care is a valuable resource to increase CRC screening through education.

## Data Availability

As the data sampled from this project involved the qualitative analysis of transcripts recorded during the meetings with participants, this has remained private and not accessible to the public. If there are concerns about the quality or replicability of this study, please contact the first author at ejkeiser@wisc.edu to discuss how this could be reviewed.
